# Effect of nutritional intervention in child health care on improving growth and development and disease prevention of infants

**DOI:** 10.12669/pjms.40.3.7720

**Published:** 2024

**Authors:** Juan Du, Feiyan Huang, Zhanghua Tang

**Affiliations:** 1 Juan Du, Department of Pediatrics, The Affiliated Hospital of Southwest, Medical University, Sichuan Clinical Research Center for Birth Defects, Luzhou 646000, Sichuan, China; 2 Feiyan Huang, Department of Pediatrics, The Affiliated Hospital of Southwest, Medical University, Sichuan Clinical Research Center for Birth Defects, Luzhou 646000, Sichuan, China; 3 Zhanghua Tang, Department of Pediatrics, The Affiliated Hospital of Southwest, Medical University, Sichuan Clinical Research Center for Birth Defects, Luzhou 646000, Sichuan, China

**Keywords:** Infants, Child Health, Nutrition Intervention, Growth and Development, Disease Prevention, Intervention Effect

## Abstract

**Objective:**

To explore the outcome of nutritional intervention in child health care on infant growth and disease prevention.

**Methods:**

It was a retrospective study. Ninty-two infants who received child health intervention in The Affiliated Hospital of Southwest Medical University from September 2020 to June 2022 were selected as the research objects. According to the random number table method, they were divided into control group (46 cases, routine health intervention) and observation group (46 cases, nutritional intervention based on the control group) using the sealed envelope system. The growth and development, nutritional diseases and guardian satisfaction of the two groups were compared.

**Results:**

The scores of the observation group were higher than those of the control group in gross motor, fine motor, language and individual-social aspects. The total incidence of nutritional diseases in the observation group (2.17%) was lower than that in the control group (17.39%); In terms of total satisfaction rate, the observation group (100.00%) was higher than the control group (86.96%), with a statistical difference (*P*<0.05).

**Conclusion:**

Applying nutrition intervention to child health care plays an important role in maintaining the healthy growth and development of infants and reducing the incidence of nutritional diseases such as anemia and rickets. It needs to be promoted in clinical practice.

## INTRODUCTION

Child health care, generally, refers to the physical and psychological health guidance for children aged 12 to 13 and below. It aimed to ensure that children are in a healthier state of growth and development, reduce the risk of infectious diseases, acute nephritis and other common childhood diseases and accidents. It also guarantee the healthy growth of children.^1^,^2^ With the promotion of the concept of good prenatal and postnatal care in China, families of children begin to think more seriously about child health care, and maintain children aged 13 and below in a more stable and healthy growth state by learning scientific education, feeding methods, taking children for vaccination on a regular basis and other measures.^3^,^4^

Nevertheless, in terms of the actual growth and development of children, diet will have a direct impact on their physical and intellectual development. If children suffer from malnutrition due to dietary problems, diseases such as muscular atrophy, physical backwardness, and brain tissue damage will be at increased risk of occurrence, seriously affecting children’s immunity, water and electrolyte balance, and even life-threatening in serious cases.^5^,^6^ Nutrition intervention can provide more scientific and appropriate dietary guidance according to the normal growth and development of children of all ages and the nutrition needed by the body. In view of this, in this study, nutrition interventions were intended to be applied to daily health care to improve the health care effect of infants.^7^

## METHODS

It was a retrospective study. Ninty-two infants who received child health intervention in The Affiliated Hospital of Southwest Medical University from September 2020 to June 2022 were selected as subjects.

### Ethical Approval:

The study was approved by the Institutional Ethics Committee of The Affiliated Hospital of Southwest Medical University, Sichuan Clinical Research Center for Birth Defects(No.:KY2020171; date: July 24, 2021), and written informed consent was obtained from all participants’ guardians.

### Inclusion criteria:

- Infants<3 years old.
- Infants with complete basic information. (This refers to various physical indicators such as height, weight, etc. of the baby.)
- Infants who were accompanied by a guardian and have signed an informed notice.

### Exclusion criteria:

- Infants with severe congenital diseases.
- Premature infants.
- Infants who withdrew from the study halfway/lost to follow-up cases.
- Who were cases and who were control?
- What randomized method was applied?
- Did the cohort had any specific coding system?

According to the random number table method, all infants were divided into the control group (46 cases, routine health intervention) and observation group (46 cases, nutritional intervention based on the control group) using the sealed envelope system. No significant difference was observed in age, body weight, gender composition and other basic data between the two groups (*P*>0.05). Table-I.

**Table-I:** Comparison of basic data between the two groups (*χ̅*±*S*, n).

Group	Number of cases	Age (month)		Bodyweight (kg)		Gender	

		Age range	Average age	Range	Average	Male	Female
Observation group	46	2-31	16.59±2.91	3.3-16.0	8.65±4.07	27 (59.70%)	19 (41.30%)
Control group	46	2-34	17.94±3.57	3.8-17.1	10.45±4.78	29 (63.04%)	17 (36.96%)
*χ^2^*/*t*	-	1.980		1.945		0.183	
*P*	-	0.051		0.055		0.669	

All infants in the control group underwent routine health care interventions, including timely instruction on the correct feeding method after the lying-in woman gave birth, notification of the approximate number of feedings and the amount of feeding, as well as instruction to family members on infant feeding related health issues and emergency management measures that could be implemented after adverse events such as milk choking. Moreover, family members should have been instructed to do the daily cleaning of infants, change diapers regularly, and a health knowledge manual related to the growth and development of infants should be distributed to family members. Infants with obvious physical and intellectual abnormalities needed to be sent to the hospital for medical treatment. In the observation group, nutritional interventions were performed on the basis of the control group, and the specific nursing was as follows:

### Strengthening of health education:

Recorded in detail the basic information of infants, including the date of birth, weight at birth, mode of birth, feeding conditions and guardian contact information, etc., and established corresponding electronic archives of nutrition and health. On a monthly basis, guardians were organized to carry out health education on infant nutrition feeding through special lectures, infant feeding activities or training. In the process of infant feeding activities and training, the instructor should promptly point out the mistakes of the guardian’s feeding methods, including feeding gestures and feeding frequency, and demonstrate scientific and correct feeding methods. Every Friday, one-on-one consultation was conducted for the guardians in turn, and the feeding situation of infants were questioned in detail, and scientific guidance was given, and the guardians were told to timely inquire if there was any doubt.

### The intervention of guardian’s feeding emotion:

The dietary intake of infants had a great impact on their physical and intellectual growth and development. Some guardians have suffered from negative emotions such as excessive worry, tension and anxiety, affecting the feeding of infants. To this end, health care staff were required to strengthen the communication and exchanged with guardians, informed guardians that no damage would cause to infants if they were fed scientifically according to health guidance, and given psychological intervention appropriately to help guardians relax.

### Nutrition diet nursing:

It was necessary to clarify the physical growth and development of infants regularly, measure and recorded the height and weight of infants every three months, and inquired in detail about the feeding conditions of infants at this stage. According to the dietary preferences of infants, formulated targeted dietary plans, formulated corresponding recipes, informed the guardian of specific cooking methods of relevant food, improved the acceptance of infants, improved partial diet, and maintained the nutritional balance of daily food intake. In addition, guardians should have been instructed to appropriately strengthen the intake of trace elements in infants, such as milk, soy products, and try to avoid the intake of candy, chocolate, potato chips and other foods. The health intervention and study lasted for six months in both groups.

### Observation indicators:

Comparison of growth and development of the two groups: The Gesell Developmental Diagnostic scale was utilized to assess the growth and development of subjects in four aspects: gross motor, fine motor, language, and personal-social before and after intervention (six months after intervention), with scores of 0-122, 0-107, 0-142, and 0-132 points, respectively. The results showed that the scores were positively correlated with good growth and development. The scores were positively correlated with good growth and development. Comparison of nutritional diseases between the two groups: Statistics were made on the number of infants who developed anemia, rickets, indigestion and other symptoms after the intervention began. Comparison of guardians’ satisfaction between the two groups: Statistics were worked out on the guardians’ satisfaction of the two groups six months after the intervention.

Guardians’ satisfaction was shown on three scales. Very satisfied: the growth and development of infants were within the normal index, without anemia, rickets and other nutritional diseases attack; Satisfied: infants with slight growth retardation, or mild anemia, rickets and other nutritional diseases attack or disease tendency, but no significant impact; Dissatisfied: infants had obvious growth retardation, or severe anemia, rickets and other nutritional diseases, which had a great impact on infants’ physical and mental health. Total satisfaction rate = (very satisfied + satisfied)/Total cases ×100%.

### Statistical Analysis:

SPSS20.0 software was utilized for statistical analysis of the data. t and “*x̅*±*S*” were used to represent measurement data, two independent sample t-test was used for comparison between groups, paired t test was used to analyze data within groups, *x^2^* and % were used to represent count data. *P*<0.05 indicates with a statistically significant difference.

## RESULTS

There was no difference in pre-intervention scores between the two groups (*P*>0.05). In terms of gross motor, fine motor, language and individual society, the score of the observation group was higher than that of the control group after intervention (*P*<0.05). Table-II

**Table-II:** Comparison of growth and development of the two groups (*χ̅*±*S*, points).

Group	Number of cases	Gross motor		Fine motor		Language		Individual-society	

		Before intervention	After intervention	Before intervention	After intervention	Before intervention	After intervention	Before intervention	After intervention
Observation group	46	90.01±3.82	107.21±5.32*	84.78±4.02	96.84±4.89*	89.89±3.54	106.85±5.94*	90.42±3.86	106.92±5.83*
Control group	46	89.93±3.88	97.32±4.23*	85.02±4.09	92.85±4.37*	89.94±3.52	98.85±4.73*	91.01±3.99	99.03±5.02*
*t*	-	0.100	9.869	0.284	4.126	0.068	7.146	0.721	6.956
*P*	-	0.921	0.001	0.777	0.001	0.946	0.001	0.473	0.001

***Note:***

* P<0.05 compared with before intervention.

The total incidence of nutritional diseases in the observation group (2.17%) was lower than that in the control group (17.39%), with a statistical difference (*P*<0.05). Table-III

**Table-III:** Comparison of nutritional diseases between the two groups [n, (%)].

Group	Number of cases	Anemia	Rickets	Indigestion	Total incidence
Observation group	46	1 (2.17%)	0	0	1 (2.17%)
Control group	46	5 (10.87%)	1 (2.17%)	2 (4.35%)	8 (17.39%)
*χ^2^*	-				4.434
*P*	-				0.035

In terms of total satisfaction rate, the observation group(100.00%) was higher than the control group (86.96%), with a statistical difference (*P*<0.05). Table-IV

**Table-IV:** Comparison of satisfaction between the two groups [n, (%)].

Group	Number of cases	Very satisfied	Satisfied	Dissatisfied	Total satisfaction rate
Observation group	46	25 (54.35%)	21 (45.65%)	0	46 (100.00%)
Control group	46	15 (32.61%)	25 (54.35%)	6 (13.04%)	40 (86.96%)
*χ^2^*	-				4.457
*P*	-				0.035

## DISCUSSION

In this study, nutrition intervention was intended to be applied to daily health care to improve the effect of infant health care and maintain the healthy growth of infants. It could be known from the results of this study that (1): in terms of gross motor, fine motor, language and individual society, the score of the observation group was higher than that of the control group after intervention(*P*<0.05), suggesting that nutritional intervention boasted to maintain the infants in a more normal and healthy growth and development state. To explain this, the nutritional intervention adopted in this study covers a series of measures, all of them could improve the guardians’ cognition of scientific infant feeding, make them aware of the impact of poor feeding methods on the growth and development of infants, and improve their attention to scientific infant feeding.^8^,^9^

At the same time, one-to-one consultation was conducted once a week to clarify the specific feeding situation of infants in detail and provided timely scientific guidance to guardians on scientific feeding, which had a positive impact on improving the standardization of scientific feeding of infants and ensuring their daily nutritional intake.^10^,^11^ Furthermore, appropriate emotional interventions were also given to the guardians to avoid excessive fear and other negative emotions, this measure played a crucial role in improving the intimacy between guardians and infants and promoting the development of the physical and mental health of infants.^12^,^13^ Therefore, nutrition intervention in addition to routine health care was more conducive to maintaining healthy growth and development of infants than routine health care alone. The total incidence of nutritional diseases in the observation group(2.17%) was lower than that in the control group(17.39%) (*P*<0.05), suggesting that nutritional intervention was beneficial to reduce the incidence of nutritional diseases.

To explain this, in addition to the nutritional intervention implemented in this study, infants were also given nutritional dietary care. Based on the regular understanding and testing of the growth and development of infants, a targeted diet plan is developed and guardians were informed of the cooking methods of relevant foods, so as to improve the acceptance of relevant foods by infants and maintain balanced nutrition.^14^ Guardians were instructed to strengthen the monitoring of the intake of milk and soy products of infants to ensure the intake of trace elements in infants. Targeted dietary guidance could timely improve the daily nutrition intake structure of infants, which was of great significance to reduce the incidence of nutritional diseases.

In terms of overall satisfaction, the observation group (100.00%) was higher than the control group (86.96%) (*P*<0.05). This was because the adoption of nutritional intervention was beneficial to the reduction of the incidence of nutritional diseases in infants and the effective maintenance of physical and mental health, and had a positive impact on promoting the healthy growth of infants. Meanwhile, the satisfaction of guardians to the relevant health care intervention also showed a significant upward trend.

Child health care is proposed to facilitate children to maintain a more normal and stable state of development and make their development within the normal index through health guidance, so as to achieve the principle of good prenatal and postnatal care.^15^,^16^ Infancy is an important stage of the physical and mental development of the human body. During this stage, the growth and development of the human body change greatly, the maturation and perfection of various tissues and organs vary, and the nutritional requirements of different growth and development stages are also quite different.^17^,^18^ In other words, infants are vulnerable to malnutrition if they are not fed scientifically and rationally, which will directly affect their physical and intellectual development.^19^,^20^

### Limitations of the study:

However, our study still had some limitations, such as a smaller sample size, shorter period of follow-up, etc., which may produce a certain impact on the level of evidence of this study. Findings in this study were expected to be confirmed through further research based on a long-term follow-up with larger sample size.

## CONCLUSIONS

Applying nutrition intervention to child health care may play an important role in maintaining the healthy growth and development of infants and reducing the incidence r of nutritional diseases such as anemia and rickets. It is worthy of popularization and application.

### Authors’ Contributions:

**JD:** Designed this study, prepared this manuscript, are responsible and accountable for the accuracy and integrity of the work.

**ZT:** Collected and analyzed clinical data.

**FH:** Data analysis**,** significantly revised this manuscript.
